# Sex differences in advance directives and their clinical translation among critically ill adults: results from the ADVISE study

**DOI:** 10.1186/s13613-025-01518-z

**Published:** 2025-07-14

**Authors:** Simon A. Amacher, Sira M. Baumann, Paulina S. C. Kliem, Dominik Vock, Yasmin Erne, Pascale Grzonka, Sebastian Berger, Martin Lohri, Sabina Hunziker, Caroline E. Gebhard, Mathias Nebiker, Luca Cioccari, Raoul Sutter

**Affiliations:** 1 Intensive Care Unit, Department of Acute Medicine, University Hospital Basel, Basel, Switzerland; 2 Department of Anesthesiology and Intensive Care Medicine, Medical Center-University of Freiburg, Faculty of Medicine, University of Freiburg, Freiburg im Breisgau, Germany; 3 Department of Intensive Care Medicine, Kantonsspital Aarau, Aarau, Switzerland; 4 Medical Communication and Psychosomatic Medicine, University Hospital Basel, Basel, Switzerland; 5 Medical Faculty of the University of Basel, Basel, Switzerland; 6 Medical Faculty of the University of Bern, Bern, Switzerland; 7 Department of Neurology, University Hospital Basel, Basel, Switzerland

**Keywords:** Advance directives, Ethics, Patients’ wills, Intensive care, Cohort study, End-of-life, Sex differences

## Abstract

**Background:**

Advance directives (ADs) are legally binding documents outlining individual preferences for medical care in the event of incapacitation. Evidence regarding their significance and implementation in critical care is scarce. Thus, this retrospective cohort study assesses sex differences in ADs’ frequency, content, clinical translation, and associated outcomes in critically ill adults. The study was performed in two interdisciplinary tertiary Swiss intensive care units (ICUs). It included patients with ADs treated in the ICUs for > 48 h. The primary endpoint was the frequency of ADs. Secondary endpoints included the content of ADs, sex differences in baseline and treatment characteristics, the clinical implementation of ADs, and in-hospital outcomes.

**Results:**

5242 patients were treated for > 48 h in the ICUs, of which 313 (6.0%) had ADs (124 females [6.8% of 1813 females] and 189 males [5.5% of 3429 males], p = 0.054). No sex-related differences were observed regarding baseline characteristics except that females with ADs were more frequently single, divorced, or widowed (57% vs. 37%, p = 0.001), more frequently had acute stroke as main diagnosis (13% vs. 3%, p = 0.001), and more often refused cardiopulmonary resuscitation (CPR) (42% vs. 25%, p = 0.002) than males with ADs. In multivariable analyses, female sex was associated with refusing CPR independent of relationship status. Compared to males, females’ ADs were more frequently violated (24% vs. 10%, p < 0.001), primarily by receiving unwanted treatments (24% vs. 8%, p < 0.001) and/or undesired ICU admission (10.5% vs 2.1%, p = 0.002). Despite these differences, treatment adaptations during intensive care, in-hospital outcomes, and discharge destinations did not differ between sexes.

**Conclusions:**

This study revealed sex disparities in the content and translation of ADs between females and males admitted to ICUs. Females’ ADs were more frequently violated, indicating a potential sex bias in the interpretation and translation of ADs in critical care. Clinicians must remain vigilant against violations of ADs and strive to deliver equitable care. Further prospective research is needed to investigate the causes of disparities in ICU end-of-life decision-making, integrating both qualitative and quantitative measures, to ensure equal treatment for all patients, regardless of sex or gender.

**Graphical Abstract:**

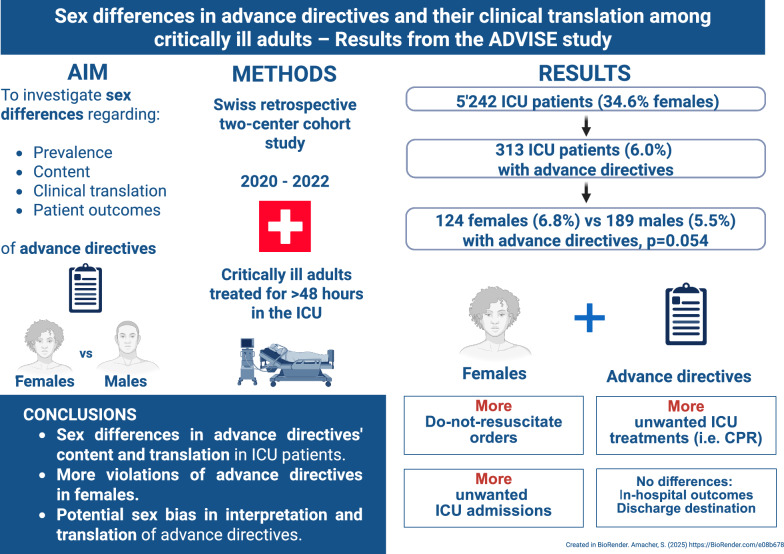

**Supplementary Information:**

The online version contains supplementary material available at 10.1186/s13613-025-01518-z.

## Background

Advance directives (ADs) are legally binding documents that outline an individual’s preferences for medical care in the event of incapacitation [1]. However, consistency, evaluation, and clinical translation remain suboptimal. The latter is reflected by a vastly heterogeneous prevalence of ADs among critically ill patients according to a systematic review of 17 studies encompassing almost 150,000 patients [2]. This study revealed a prevalence of written ADs between 2.6% in Northern and Southern European and 49% in North American intensive care units (ICUs) [2]. Such low numbers might come with increasing costs and unwanted treatments, as strictly followed ADs are associated with increased do-not-resuscitate orders, care limitations, and shorter ICU stays, which may result in lower healthcare expenses [2]. The situation is further aggravated, as less than 10% of healthcare providers systematically screen for ADs [2]. These effects might be significant in the face of modern high-technology critical care, with continuously increasing ICU survival rates leading to higher numbers of patients with long-term disabilities [3–6]. This can impose a significant psychological and economic burden on individuals, families, and society [3–6]. However, previous studies regarding the financial and resource-sparing effects of ADs in critical care have provided mixed results, and their efficacy is questionable [7–13].

An increasing body of evidence suggests significant sex-related differences when it comes to the provision of intensive care [14, 15] and the content and clinical translation of ADs [16]. Furthermore, some studies reported that treatment limitations are more prevalent in critically ill females [14, 17–20]. Also, female sex might be a risk factor for early withdrawal of life-sustaining therapies [20–23]. However, data on sex-related differences in prevalence, content, and translation of ADs in critical care is scarce [2].

Thus, this two-center study assessed differences between males and females regarding the prevalence, content, clinical translation, and associated patient outcomes of ADs in critically ill adults.

## Methods

### Design and ethics

This retrospective cohort study was performed at the interdisciplinary ICUs of the Cantonal Hospital of Aarau and the University Hospital of Basel in Switzerland, both tertiary academic medical centers. All data were collected as part of the ongoing **ADVISE** (**A**dvance **D**irective **I**mplementation and **S**cientific **E**valuation) study project, registered at ClinicalTrials.gov (NCT04348318). The project was approved by the ethics committee of Northwestern and Central Switzerland—EKNZ (https://www.eknz.ch/) in March 2020 (BASEC No. 2020-00584). The study’s conduct and reporting followed the STROBE guidelines [24].

### Primary and secondary endpoints

The primary endpoint was the frequency of ADs. Secondary endpoints were the content of ADs, differences between males and females regarding baseline, clinical, and treatment characteristics, clinical implementation of ADs, and in-hospital outcomes.

### Data assessment and extraction

From January 2020 to December 2022, all consecutive adult patients (i.e., ≥ 18 years) treated for > 48 h in the ICUs of both centers were retrospectively enrolled in the study. Their clinical data was collected from digital medical records and entered into a password-protected online assessment platform (Research Electronic Data Capture) into a database designed for this study [25]. For the database’s design, the key research group members (RS, SMB, SAA) thoroughly evaluated all available Swiss AD templates and decided which parameters to extract in a shared decision-making discussion.

Initially, the primary individual responsible for data extraction (SMB) received training and supervision from the project leader (RS) to ensure the accuracy of data extraction. Subsequently, SMB provided training and oversight to the other data extraction team members (PSCK, DV, and YE). Data extraction was performed individually. In cases of uncertainty concerning correct data, the project leader was consulted to make a final decision. Before data analysis, the principal investigator (RS) conducted an independent quality control assessment to verify the consistency and accuracy of the data.

Data on patient demographics, admission characteristics (including main diagnosis), severity of illness (quantified as outlined below), level of consciousness, treatment interventions (including respiratory support, mechanical and medical hemodynamic support, medication administration, fluid administration, blood products, antibiotics, nutrition, pain medication), and complications during intensive care were collected as reported in the patient records. We further reviewed patients’ medical records to identify clinical notes and reports detailing any adjustments made to their intensive care treatment. This included information on the timing and type of withdrawal or discontinuation of life-prolonging treatments, changes in cardiopulmonary resuscitation (CPR) status, and decisions to withhold specific life-prolonging measures. Inpatient outcomes included death, the patients’ return to their premorbid neurologic function at discharge, and discharge destination.

### Local jurisdiction

In Switzerland, written ADs are legally binding instruments, and their use is regulated by the Swiss Society for Medical Sciences (SAMW) guidelines [26]. The following formal criteria must be fulfilled for the ADs’ validity:

- Decision-making capacity: The individual must clearly understand the ADs’ content and the potential consequences of the directives provided.
- Formalities: The document requires either the patient’s signature or a notarial certification for legal validity.
- Content: The ADs’ content must comply with local laws, and the ADs cannot request unlawful actions (e.g., active euthanasia).

The treating physicians are obliged to adhere to the ADs unless there is a well-founded suspicion that the above-specified formal requirements are not fulfilled.

In Switzerland, ADs serve two main purposes in the event of a patient’s incapacitation: first, they designate a healthcare proxy for decision-making. Second, they outline the patient's preferred and refused medical treatments in case of severe illness.

The ADs’ stipulations supersede the preferences of the healthcare proxy and medical team when the formal requirements for the AD’s validity are met. Nevertheless, requests for non-beneficial or illegal treatments or interventions are inadmissible. The proxy decisions must align with the patient’s wishes and best interests, guided by the written ADs. This is particularly important if the AD is outdated or the medical condition necessitates a different course of action. However, caregivers must consult the proxy (if defined) for shared decision-making in such specific situations. Medical expertise should be integrated into the shared decision-making process, particularly in cases where outdated ADs or a patient's changing condition necessitates changes to the treatment plan. However, healthcare proxy consultation remains essential for shared decision-making in such circumstances.

The Swiss Medical Association’s (FMH) official AD template comprises two sections: a general statement regarding values for life and death and specific instructions regarding medical procedures and treatment goals [27].

### Assessment of illness severity

We assessed illness severity upon ICU admission using the Acute Physiology And Chronic Health Evaluation (APACHE) II [28, 29], the Charlson Comorbidity Index [30], and the Good Outcome Following Attempted Resuscitation (GO-FAR) score [31]. Due to the study’s retrospective design, all scores were retrospectively calculated based on a review of medical charts. The online Supplementary Material provides further details regarding the individual scores.

### Assessment of advance directives

All ADs were reviewed to collect information on preferences regarding end-of-life (EoL) care. This included the issue date, the presence and validity of the signature, the situations under which the ADs would apply, preferences regarding life-prolonging measures and CPR, preferences for ICU and hospital admission, preferences for best supportive care, desired or undesired treatments (e.g., artificial nutrition, hydration, pain management, specific medications, surgery, mechanical ventilation, hemodynamic support, renal replacement therapy), and any other relevant information. Four reviewers (S.M.B., D.V., P.SC.K, and Y.E.) extracted data from the ADs under continuous mutual consultation to guarantee high data quality.

### Statistics

Baseline characteristics, clinical and treatment characteristics, and characteristics related to the clinical translation of AD content were summarized with descriptive statistics. Continuous variables were presented as medians with interquartile ranges, while categorical variables were expressed as counts and percentages. Patients were categorized based on their biological sex category. Between males and females, we conducted univariable comparisons of proportions using the Chi-square or Fisher’s exact test. A Shapiro–Wilk test was performed to distinguish between normally and not normally distributed continuous variables. Normally distributed variables were then analyzed using the Student’s *t-*test, whereas non-normally distributed variables were analyzed using the Mann–Whitney *U* test. The significance level for univariable comparisons was adjusted using the Bonferroni correction and set at a two-sided p-value of ≤ 0.01 or ≤ 0.007, depending on the number of comparisons for each endpoint.

Multivariable logistic regression analysis was performed to identify characteristics independently associated with refusing CPR or violation of ADs. The Hosmer–Lemeshow Chi-square goodness-of-fit test was used to assess the calibration of the multivariable logistic regression models [32]. Statistical analyses were conducted using Stata 16.1 (StataCorp, College Station, TX, USA).

## Results

### Primary endpoints

Of the 5242 patients treated for > 48 h in the ICUs, 313 (6.0%) had ADs. ADs could be identified in 6.8% of 1813 females (n = 124) and in 5.5% of 3429 males (n = 189), p = 0.054. Of the 313 patients with ADs, 124 were female (39.6%). The proportion of males and females in both centers was similar, as of 68 patients in Aarau, 26 were female [38.2%], and of 245 patients in Basel, 98 were female [40%], p = 0.889). Please refer to Supplementary Fig. 1 for the detailed study flow chart.

### Secondary endpoints

#### Sex-related differences in baseline characteristics

Females with ADs were more likely to be single, divorced, or widowed (57% vs. 37%, p = 0.001, i.e. relationship status) and were more frequently admitted to the ICU with acute stroke as a primary diagnosis (13% vs. 3%, p = 0.001). The remaining variables, including age, confession, level of consciousness, care dependency, prior ICU treatment, illness severity scores, and the remaining admission diagnoses, did not exhibit significant sex disparities, Table 1. Interestingly, in 23.6% of patients ADs were unavailable at ICU admission but recognized later during the ICU stay without differences between sexes, Table 1.

**Table 1:** Univariable comparisons of demographics and baseline characteristics between critically ill males (n = 189) and females (n = 124) with advance directives treated in the ICUs for > 48 h (n = 313)

Characteristics	Females (n = 124)		Males (n = 189)		p-value*
Baseline characteristics	n/median	%/IQR	n/median	%/IQR	
Age (years; median, IQR)	72	64–81	72	66–78	0.408
Relationship status (single, divorced or widowed) (n, %)	70	56.5	70	37.0	**0.001**
Care dependency prior admission (n, %)	29	23.4	44	23.3	0.983
Previous ICU stay (n, %)	44	35.5	87	46.0	0.064
**Confession (n, %)**					
None	49	39.5	74	39.2	0.369
Roman catholic	28	22.6	56	29.6	
Reformed	33	26.6	47	24.9	
Others	9	7.3	6	3.2	
Unknown/not stated	5	4.0	6	3.2	
**Main admission diagnosis (not mutually exclusive; n, %)**					
Postoperative care	21	16.9	29	15.3	0.707
Sepsis	17	13.7	23	12.2	0.690
Heart failure	8	6.5	20	10.6	0.231
Stroke	16	12.9	6	3.2	**0.001**
COVID-19	7	5.7	15	7.9	0.504
Pneumonia	3	2.4	13	6.9	0.114
Epileptic seizures	6	4.8	7	3.7	0.773
Myocardial infarction	2	1.6	10	5.3	0.134
Trauma	4	3.2	7	3.7	NA
Chronic obstructive pulmonary disease exacerbation	2	1.6	8	4.2	NA
Gastrointestinal bleeding	2	1.6	4	2.1	NA
Acute liver failure	3	2.4	3	1.6	NA
Delirium	1	0.8	3	1.6	NA
Ketoacidosis	0	0.0	3	1.6	NA
Encephalitis/meningitis	3	2.4	0	0.0	NA
Intoxication	1	0.8	1	0.5	NA
Pulmonary embolism	0	0.0	1	0.5	NA
Arrhythmia	1	0.8	0	0.0	NA
Others	27	21.8	36	19.0	NA
**Illness severity scores (median, IQR)**					
Charlson Comorbidity Index	5	3–6	5	4–7	0.016
APACHE II (32 missing values)	29	23–33	30	25–35	0.079
GO-FAR	16	6–24	14	7–22	0.857
**GCS at ICU admission (median, IQR)**	9	3–14	8	3–14	0.584
**Availability and recognition of advance directives in the ICU (n, %)**					
Advance directives available and recognized at ICU admission	94	75.8	145	76.7	0.852
Advance directives available and recognized later during ICU stay	30	24.2	44	23.3	
**Advance directives (n, %)**					
Patients with advance directives regarding life-prolonging measures	114	91.9	177	93.7	0.561
Patients refusing life-prolonging measures	61	49.2	68	36.0	0.020
Patients with advance directives regarding cardiopulmonary resuscitation	89	71.8	132	69.8	0.714
Patients refusing cardiopulmonary resuscitation	52	41.9	48	25.4	**0.002**

*IQR* interquartile range, *ICU* intensive care unit, *COVID-19* Coronavirus disease 2019, *GCS* Glasgow Coma Score, *NA* not applicable

Charlson Comorbidity Index (range 0–37) [30]; APACHE II = Acute Physiology and Chronic Health Evaluation II (Range 0–71) [28, 29]; GO-FAR = Good Outcome Following Attempted Resuscitation (range -15–76) [31]; Glasgow Coma Score (range 3–15) [33]

^*^Level of significance adjusted using the Bonferroni correction for multiple comparisons and set at a significance level at a two-sided p-value of ≤ 0.007 due to the Bonferroni correction

^*^Bold font numbers indicate statistical significance after Bonferroni correction

#### Advance directives’ content

**Fig. 1:**
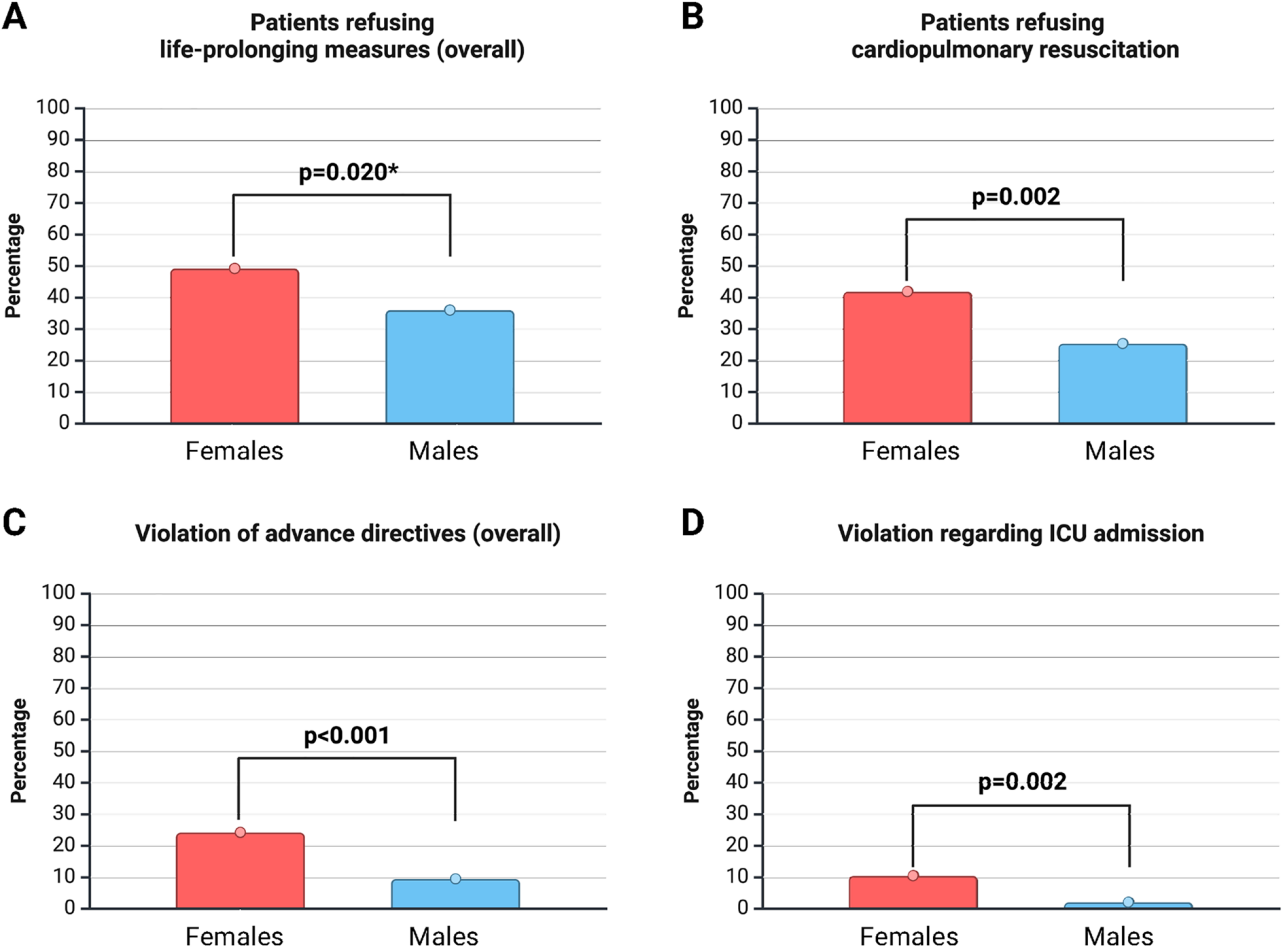
Sex differences regarding preferences for life-prolonging measures and violations of advance directives during critical care. *not reaching the statistical significance at a two-sided p-value of ≤ 0.007 after Bonferroni correction for multiple comparisons. **A** Prevalence of life-prolonging measures refusal (overall). **B** Prevalence of cardiopulmonary resuscitation refusal. **C** Prevalence of advance directives’ violation (overall). **D** Prevalence of ICU admission violation

We observed higher refusal rates for life-prolonging measures in general among females, however, without meeting the conventional level of statistical significance after Bonferroni correction (refusal of life-prolonging measures females vs males, 49.2% vs. 36.0%, p = 0.020). Nevertheless, a significantly higher proportion of females specifically refused CPR (42% vs. 25%, p = 0.002), Table 1, Fig. 1.

Multivariable analysis revealed that female sex was an independent predictor of CPR refusal, even after accounting for relationship status (married, in partnership, single, divorced, or widowed). While females were twice as likely as males to refuse CPR (odds ratio [OR] 2.01, 95% confidence interval [CI] 1.23–3.28, p = 0.006), relationship status was not significantly associated with CPR refusal (OR 0.74, 95%CI 0.45–1.2, p = 0.221).

#### Sex-related differences regarding clinical translation of ADs

Univariable comparisons regarding the clinical translation and treatment adaptation following patients’ ADs revealed significant disparities between males and females, as outlined in Table 2. During intensive care, females’ ADs were violated more frequently than males’ (24% vs. 10%, p < 0.001; Fig. 1). The most common way of AD violations was the provision of unwanted treatments (24% vs. 8%, p < 0.001). In particular, females were significantly more often admitted to the ICU despite an explicit refusal stated in their AD (10.5% vs. 2.1%, p = 0.002), Table 2, upper half. Even when excluding patients whose ADs were unavailable at ICU admission, females still experienced a higher rate of AD violations (21% vs. 8%, p = 0.006; 6% vs. 1%, p = 0.007; Table 2, lower half). Interestingly, the provision of unwanted treatments was found to be independent of relationship status, Supplementary Table 1.

**Table 2:** Univariable comparisons regarding the clinical translation and treatment adaptation of critically ill males (n = 189) and females (n = 124) with advance directives treated in the ICUs for > 48 h (n = 313)

Characteristics	Females (n = 124)		Males (n = 189)		p-value*
Violation of advance directives during intensive care (n, %)	n/median	%/IQR	n/median	%/IQR	
Violation of patients’ advance directives (not mutually exclusive)	30	24.2	18	9.5	**< 0.001**
Violation regarding life-prolonging measures	17	13.7	10	5.3	0.013
Violation regarding ICU admission	13	10.5	4	2.1	**0.002**
Violation regarding mechanical ventilation	9	7.3	7	3.7	0.193
Violation regarding artificial nutrition	6	4.8	8	4.2	0.787
Violation regarding CPR	7	5.7	4	2.1	0.121
Violation regarding surgery	3	2.4	1	0.5	0.305
Violation regarding treatment of infections	3	2.4	0	0.0	0.061
Violation regarding sedation	1	0.8	0	0.0	NA
Violation regarding organ donation (not performed despite wish)	1	0.8	0	0.0	NA
Violation regarding administration of blood products	0	0.0	1	0.5	NA
Violation regarding desired place of death	0	0.0	0	0.0	NA
Violation regarding mechanical hemodynamic support	0	0.0	0	0.0	NA
Violation regarding renal replacement	0	0.0	0	0.0	NA
Violation regarding pain management	0	0.0	0	0.0	NA

Violation of advance directives despite recognized at ICU admission (recognized in 239 patients)	Females with advance directives recognized at ICU admission (n = 94)		Males with advance directives recognized at ICU admission (n = 145)		p-value*
	n/median	%/IQR	n/median	%/IQR	
Violation of patients’ advance directives (not mutually exclusive; n, %)	20	21.3	12	8.3	**0.006**
Violation regarding life-prolonging measures (n, %)	11	11.7	6	4.1	0.038
Violation regarding ICU admission (n, %)	10	10.6	3	2.1	**0.007**
Violation regarding mechanical ventilation (n, %)	6	6.4	5	3.5	0.349
Violation regarding artificial nutrition (n, %)	4	4.3	7	4.8	1.000
Violation regarding CPR (n, %)	5	5.3	2	1.4	0.115
Violation regarding surgery (n, %)	3	3.2	1	0.7	0.303
Violation regarding treatment of infections (n, %)	2	2.1	0	0.0	0.154
Violation regarding sedation (n, %)	1	1.1	0	0.0	NA
Violation regarding organ donation (not performed despite wish; n, %)	0	0.0	0	0.0	NA
Violation regarding administration of blood products (n, %)	0	0.0	0	0.0	NA
**Treatment adaptation during intensive care**					
Treatment adaptation overall (not mutually exclusive; n, %)	45	36.3	59	31.2	0.351
Withdrawal of life-sustaining therapies (n, %)	26	21.0	37	19.6	0.764
Withholding of life-sustaining therapies (n, %)	32	25.8	44	23.3	0.610
Time of ICU admission to adaptation of life-sustaining therapies (days; median, IQR)	5.5	2.5–10.5	5	2.0–9.0	0.864
Changes of CPR status	45	36.3	59	31.2	0.351
Time of ICU admission to change of CPR status (days; median, IQR)	3	0–6	4	1–9	0.149

*IQR* interquartile range, *ICU* intensive care unit, *CPR* cardiopulmonary resuscitation, *NA* not applicable

^*^Level of significance adjusted using the Bonferroni correction for multiple comparisons and set at a significance level at a two-sided p-value of ≤ 0.01 due to the Bonferroni correction

^*^Bold font numbers indicate statistical significance after Bonferroni correction

To assess whether this difference remains significant independent of potential differences in patient management between the centers, logistic regression analyses adjusting for the two centers were performed and confirmed center independence of this result, with an increased odds of AD violation for female sex (OR 3.00, 95% CI 1.39–6.48, p = 0.005).

Table 3 provides a granular examination of the specific types of AD violations. The provision of unwanted treatments was the most common violation, while withholding desired treatments was less frequently observed.

**Table 3:** Univariable comparisons regarding the type of violation of advance directives of critically ill males (n = 189) and females (n = 124) treated in the ICUs for > 48 h (n = 313)

Violation of advance directives during intensive care (n, %)	Females (n = 124)		Males (n = 189)		p-value*
**Treatment despite refusing such according to advance directives**	n	%	n	%	
Unwanted treatments overall (not mutually exclusive)	30	24.2	15	7.9	** < 0.001**
Unwanted life-prolonging measures	14	11.3	10	5.3	0.080
Unwanted ICU admission	13	10.5	4	2.1	**0.002**
Unwanted mechanical ventilation	5	4.0	6	3.2	0.758
Unwanted artificial nutrition	5	4.0	8	4.2	1.000
Unwanted CPR	7	5.6	1	0.5	**0.007**
Unwanted surgery	3	2.4	1	0.5	0.305
Unwanted treatment of infections	3	2.4	0	0.0	NA
Unwanted sedation	1	0.8	0	0.0	NA
Unwanted organ donation (not performed despite wish)	0	0.0	0	0.0	NA
Unwanted administration of blood products	0	0.0	1	0.5	NA
Unwanted desired place of death	0	0.0	0	0.0	NA
Unwanted mechanical hemodynamic support	0	0.0	0	0.0	NA
Unwanted renal replacement	0	0.0	0	0.0	NA
Unwanted pain management	0	0.0	0	0.0	NA
**No treatment despite consent to such according to advance directives**					
Unwanted withholding of treatments overall (not mutually exclusive)	3	2.4	3	1.6	0.684
Unwanted withholding of life-prolonging measures	3	2.4	0	0.0	NA
Unwanted withholding of mechanical ventilation	4	3.2	1	0.5	NA
Unwanted withholding of artificial nutrition	1	0.8	0	0.0	NA
Unwanted withholding of CPR	0	0.0	3	1.6	NA
Unwanted withholding of surgery	0	0.0	0	0.0	NA
Unwanted withholding of treatment of infections	0	0.0	0	0.0	NA
Unwanted withholding of sedation	0	0.0	0	0.0	NA
Unwanted withholding of organ donation (not performed despite wish)	1	0.8	0	0.0	NA
Unwanted withholding of administration of blood products	0	0.0	0	0.0	NA
Unwanted withholding of desired place of death	0	0.0	0	0.0	NA
Unwanted withholding of mechanical hemodynamic support	0	0.0	0	0.0	NA
Unwanted withholding of renal replacement	0	0.0	0	0.0	NA
Unwanted withholding of pain management	0	0.0	0	0.0	NA

*IQR* interquartile range, *ICU* intensive care unit, *CPR* cardiopulmonary resuscitation, *NA* not applicable

^*^Level of significance adjusted using the Bonferroni correction for multiple comparisons and set at a significance level at a two-sided p-value of ≤ 0.01 due to the Bonferroni correction

^*^Bold font numbers indicate statistical significance after Bonferroni correction

#### Inpatient outcomes and discharge destinations

A comparative analysis of inpatient outcomes (in-hospital death, hospital and ICU length of stay) and discharge destination, stratified by sex category, is presented in Table 4. Due to the inclusion criteria, our cohort exhibited a substantial in-hospital mortality rate of 33.2% and a return to premorbid neurological function in only 20.1% of cases.

**Table 4:** Univariable comparisons regarding inpatient outcomes and discharge destinations of critically ill males and females treated in the ICUs for > 48 h (n = 313)

Total cohort (n = 313)	Females (n = 124)		Males (n = 189)		p-value*
In-hospital outcomes	n/median	%/IQR	n/median	%/IQR	
Hospital length of stay (days; median, IQR)	18	11–32	18	11–30	0.756
ICU length of stay (days; median, IQR)	6	3–9	5	4–10	0.800
In-hospital death (n, %)	42	33.9	62	32.8	0.845
Return to premorbid function (n, %)	21	16.9	42	22.2	0.313

Discharge destination of survivors	n	%	n	%	
Home (n, %)	16	12.9	17	9.0	0.755
Rehabilitation (n, %)	42	33.9	74	39.2	
Other hospital (n, %)	14	11.3	23	12.2	
Hospice (n, %)	10	8.1	13	6.9	

Patients with AD violation (n = 48)	Females (n = 30)		Males (n = 18)		
In-hospital outcomes	n/median	%/IQR	n/median	%/IQR	p-value*
Hospital length of stay (days; median, IQR)	17	9–31	28	8–36	0.424
ICU length of stay (days; median, IQR)	6	3–11	6	4–9	0.881
In-hospital death (n, %)	13	43.3	10	55.6	0.412
Return to premorbid function (n, %)	4	13.3	1	5.6	0.637

Discharge destination of survivors	n	%	n	%	
Home (n, %)	1	3.3	0	0.0	0.975
Rehabilitation (n, %)	10	33.3	5	27.8	
Other hospital (n, %)	4	13.3	2	11.1	
Nursing home, hospice (n, %)	2	6.7	1	5.6	

*AD* Advance directive, *IQR* interquartile range, *ICU* intensive care unit, *CPR* cardiopulmonary resuscitation

^*^Level of significance adjusted using the Bonferroni correction for multiple comparisons and set at a significance level at a two-sided p-value of ≤ 0.01 due to the Bonferroni correction

^*^Bold font indicating significance

Despite the observed differences in baseline characteristics and AD content of male and female patients, there were no significant sex-related differences in treatment adjustments during intensive care, in-hospital outcomes, or discharge destinations, Table 4.

## Discussion

This Swiss observational two-center study, which included critically ill adult ICU patients with an ICU length of stay > 48 h, investigated the frequency, clinical translation, and associated outcomes of ADs, focusing on sex-related differences. Due to the inclusion criteria, our cohort exhibited a significant in-hospital mortality of 33.2% and a return to premorbid neurological function in only 20.1% of cases.

**Fig. 2:**
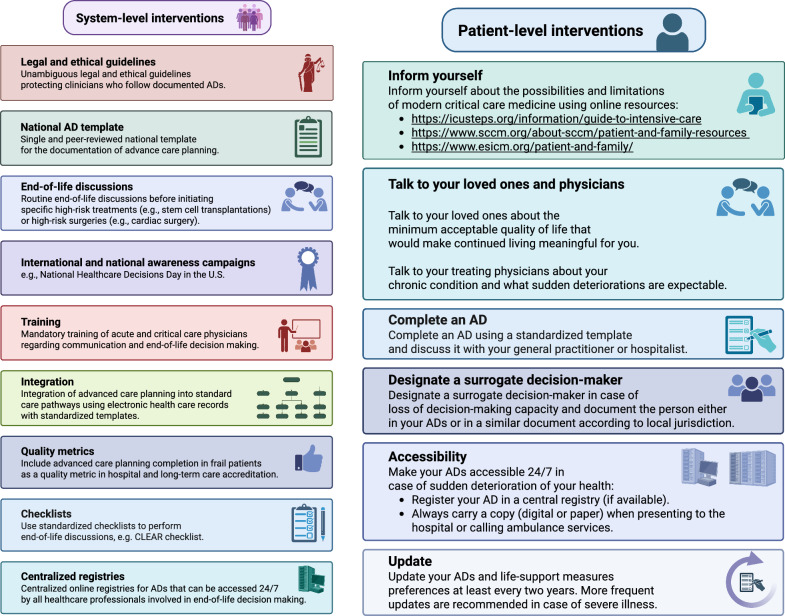
Suggested system- and patient level interventions to improve advanced care planning and end-of-life decision making. **A** System-level interventions. **B** Patient-level interventions. AD Advance directive, CLEAR Clinician–patient engagement; Learn and inform; Explore patient preferences; Assess and document; and Review advance directives Checklist [39]

Only a minority of critically ill patients had written ADs, with a borderline not significantly higher proportion of ADs in females. Interestingly, ADs were unavailable at ICU admission in approximately one-quarter of patients but were recognized later during the ICU stay. This highlights the urgent need for central AD registries and dedicated investigations regarding ADs upon ICU admission. In our view, the low availability of ADs represents just the ‘tip of the iceberg’, and we are convinced that substantial improvements in advanced care planning are urgently needed. Figure 2 suggests several patient and healthcare system interventions that could improve advanced care planning: In the US Medicaid/Medicare system, formal advance directive discussions or documented advance directives in patients aged 65 years or older are integrated into the accreditation processes of hospitals and long-term care facilities and are rewarded in specific payment systems [34]. Previous studies have demonstrated the benefits of integrating advanced care planning into routine care pathways through the use of electronic health records with a standardized documentation template [35, 36]. A particular focus should be on training future critical care physicians in end-of-life decision-making and ethical considerations. In theory, this is part of the Swiss curriculum for board-certified intensive care physicians; however, there is no specific training course for end-of-life decision-making and physician–patient communication for critical care providers. This should be integrated into critical care training as a mandatory qualification for board certification. In the United States, several states have implemented central registries for ADs and advance care planning, leading to the presence of an AD in up to 50% of deceased ICU patients or those with limitations of life-sustaining therapies [37, 38].

While most variables, including demographics, care dependency, level of consciousness, prior ICU treatment, illness severity scores, and most admission diagnoses, did not exhibit substantial sex disparities, females with ADs were more likely to be single, divorced, or widowed. This corresponds with previous research showing that elderly females are more likely to live alone than elderly males, potentially resulting in differences in EoL decision-making [40, 41].

Despite the low prevalence of ADs among our cohort of high-risk critically ill patients, we found relevant sex-associated differences in their content, use, and clinical implementation. Although females were more likely to possess ADs and refuse CPR as compared to males, their ADs were violated more frequently, particularly by involuntary ICU admission and provision of unwanted treatments. Interestingly, this was independent of relationship status. Our findings indicate that female sex may be a relevant risk factor for the receipt of unwanted intensive care treatments. Even when excluding patients whose ADs were not available at ICU admission, but recognized later during intensive care, females still experienced a higher rate of violations. This suggests that the observed differences in AD violations are not solely attributable to the availability of ADs at ICU admission but rather reflect sex-related underlying disparities in how these ADs are respected and implemented.

Previous research focusing on sex and gender-related differences in the provision of intensive care revealed that female sex is generally associated with lower ICU admission rates [14, 15, 42], and less aggressive ICU treatments, such as mechanical ventilation, renal replacement therapy, or other vital organ support, as well as a higher risk for withdrawal of life-sustaining therapies [14, 15, 21, 42–46].

The reasons are multifactorial and might be partly explained by sex and/or gender differences in attitudes, as females seem more open-minded towards EoL discussions than males [47]. Also, females tend to have a greater aversion towards life-sustaining therapies, regarding more invasive or ‘heroic’ interventions [48, 49]. This is in line with our study, as females had a two-fold higher likelihood of refusing CPR, which remained true even after adjusting for marital or partnership status, supporting the concept of sex and/or gender differences in EoL preferences.

The higher preference for a Do Not Resuscitate (DNR) order in females aligns with previous research [48, 50, 51]. In a survey of multiethnic inpatients regarding attitudes about advance care planning and dying, males and females exhibited distinct EoL preferences [16]. While males seem to prioritize functional outcomes and express concerns about healthcare system-induced harm and disempowerment, females encompass a broader spectrum of factors, including death location, and demonstrate greater trust in the healthcare system [16]. In particular, females seem to prefer a dignified death and avoid non-beneficial interventions [48]. However, there might be significant cultural differences, as studies from Europe [50, 51] and the USA [48] show a higher preference for DNR in females, while a Southeast Asian study revealed higher DNR rates in males [52].

Thus, due to various reasons, females in general receive fewer life-sustaining therapies; however, when explicitly refusing such treatments, those preferences seem to be frequently overruled, as shown in our study. Interestingly, in our study, the overruling of female ADs was independent of relationship status. On the other hand, in a US multicentric cohort study of metastatic cancer patients, males had a higher risk than females of receiving aggressive and potentially non-beneficial intensive care near death [46].

In summary, several conscious or subconscious sex-related biases seem to play a role in EoL decision-making. Despite the observed differences in the clinical translation of ADs, no significant sex disparities were found in patient outcomes or post-hospital care destinations. However, these findings need verification in multicentric prospective cohort studies.

Our study exhibits several strengths. First, the two-centric design, including two large tertiary intensive care units, gives the study high external validity. Second, many characteristics of our ICU population are similar to those of other ICU cohort studies, such as age [53, 54], sex category [54], admission diagnosis [53], and illness severity [54]. Third, the comprehensive assessment of ADs’ content by multiple investigators provides insights into the detailed individual ADs regarding medical treatment and their clinical translation, which, to the best of the author’s knowledge, has not been analyzed in ICU patients at this level so far.

Several important limitations must be considered. First, due to the study’s retrospective design, all clinical scores had to be retrospectively calculated based on medical chart review, which might have led to documentation bias. Also, due to inconsistent documentation and the retrospective study design, it was challenging to determine the exact time during the ICU stay when ADs were recognized. This might have led to the omission of patients whose ADs were recognized but not documented. However, since the availability of ADs is generally considered a significant advantage in patient-centered care, it is unlikely that the number of unrecorded or unrecognized ADs is substantial. As patients in Switzerland are not obliged to use standardized templates for their ADs, and even these templates may contain a variable amount of free text, we cannot exclude the possibility that a subjective interpretation of the free text passages has influenced our results. However, in the authors’ clinical experience, ADs written in full text without using templates that guide the author with unambiguous choices are very uncommon, and free text fields are mostly limited. Additionally, different jurisdictions and regulations in other countries may hinder the generalizability of our results.

Furthermore, the study’s design prevented us from definitively establishing whether the decision to withdraw or withhold care was solely based on patient ADs, and confounding factors may have influenced the decision-making process. Patients’ preferences can change over time. Thus, it is possible that ICU patients with decision-making capacity chose to revoke their previously documented ADs. If this occurred without being documented in the medical records, then the care provided would not be a violation of patient preferences, but an honoring of the patient’s current preferences for intensive care. Also, due to the retrospective design, our ability to investigate the reasons behind the absence of ADs was hindered. This renders the differentiation between patients who never completed an AD and those whose existing ADs were not identified, difficult. However, since our primary goal was to assess the frequency of identified ADs, this limitation did not impact our results. Since females, compared to males, tend to have a greater aversion towards life-sustaining therapies and probably more frequently possess ADs, our results might not indicate a sex and/or gender bias in the translation of ADs but rather result from treatment limitations being less likely to be honoured in the ICU. Finally, as healthcare records generally include sex, rather than gender, the current study primarily investigated sex differences. Yet the observed differences are most probably not only related to biological differences between males and females but also to sociocultural differences between females and males, i.e., gender differences. However, the concepts of sex and gender are very closely related, and we thus consider the effect to be minor.

## Conclusions

This study revealed sex disparities in the content and translation of ADs between females and males admitted to ICUs. Females’ ADs were more frequently violated, indicating a potential sex bias in the interpretation and translation of ADs in critical care. Clinicians must remain vigilant against violations of ADs and strive to deliver equitable care. Further prospective research is needed to investigate the causes of disparities in ICU end-of-life decision-making, integrating both qualitative and quantitative measures, to ensure equal treatment for all patients, regardless of sex or gender.

## Supplementary Information

Supplementary Material 1.

## Data Availability

The raw data can be obtained from the corresponding author (Raoul Sutter, MD, raoul.sutter@usb.ch) upon reasonable request.

## Abbreviations

AD: Advance Directives
ADVISE: Advance Directive Implementation and Scientific Evaluation
APACHE: Acute Physiology And Chronic Health Evaluation
CLEAR: Clinician–patient engagement; Learn and inform; Explore patient preferences; Assess and document; and Review advance directives
CPR: Cardiopulmonary resuscitation
DNR: Do not resuscitate
EoL: End-of-life
GO-FAR: Good Outcome Following Attempted Resuscitation
ICU: Intensive care unit
OR: Odds ratio
REDCAP: Research electronic data capture
STROBE: Strengthening the Reporting of Observational Studies in Epidemiology
