# Supplementary material for: Sex differences in advance directives and their clinical translation among critically ill adults: results from the ADVISE study
Source: Ann Intensive Care. 2025 Jul 14;15:94. doi: 10.1186/s13613-025-01518-z (PMC12259517; doi:10.1186/s13613-025-01518-z)
Supplement: Supplementary file 1 — Supplementary Material 1. [file 13613_2025_1518_MOESM1_ESM.docx]

**Sex differences in advance directives and their clinical translation among critically ill adults – Results from the ADVISE study**

**Supplementary Material**

**Corresponding Author:**

Raoul Sutter, MD

Intensive Care Unit and Department of Neurology

University Hospital Basel

CH-4031 Basel

Switzerland

[raoul.sutter@usb.ch](mailto:raoul.sutter@usb.ch).

Phone: +4161’265’25’25

**Supplementary methods**

*Assessment of illness severity - details*

The APACHE II (Acute Physiology and Chronic Health Evaluation II) is a scoring system (ranging from 0 to 71 points) used to assess the severity of illness in critically ill patients admitted to ICUs. It calculates a score based on physiological measurements, age, and chronic health conditions(23,24). Lower scores on the APACHE II score indicate a better prognosis(23,24).

The Charlson Comorbidity Index is a scoring system (ranging from 0 to 37 points) used to quantify the severity of underlying medical conditions in patients and mainly used to assess the overall health status of individuals and to predict mortality risk in various settings, including healthcare, epidemiology, and public health(25). Lower scores on the Charlson Comorbidity Index indicate a lower level of comorbidities.

The GO-FAR score (ranging from -15 to 76) is a prognostic tool specifically designed for critically ill patients who have experienced a cardiac arrest. It evaluates the likelihood of survival with a good neurological outcome based on age, underlying medical conditions, and initial heart rhythm. Lower scores on the GO-FAR indicate a better prognosis.

**Supplementary figures**

**Supplementary Figure 1. Study flow chart.**

**Supplementary table 1** Univariable comparisons regarding unwanted treatment between critically ill females and males treated in the ICUs for >48 hours with respect to marital or partnership status

| **Violation of advance directives during intensive care despite recognition at ICU admission (n, %)** | **Females in partnership**  **or married**  **(n=54)** | | **Males in partnership**  **or married**  **(n=119)** | |  |
| --- | --- | --- | --- | --- | --- |
|  | **n** | **%** | **n** | **%** | **p-value** |
| Unwanted treatments | 14 | 25.9 | 9 | 7.6 | **0.003** |
|  | **Females without partnership**  **(n=70)** | | **Males without partnership**  **(n=70)** | |  |
|  | **n** | **%** | **n** | **%** | **p-value** |
| Unwanted treatments | 16 | 22.9 | 6 | 8.6 | **0.035** |

**Supplementary references**

1. **SAMW Richtlinien Patientenverfügung** [<https://www.samw.ch/de/Ethik/Themen-A-bis-Z/Patientenverfuegung.html>]

2. **Swiss Medical Association - Advance directive** [<https://www.fmh.ch/dienstleistungen/recht/patientenverfuegung.cfm>]
